# Degradation Model of Bioabsorbable Cardiovascular Stents

**DOI:** 10.1371/journal.pone.0110278

**Published:** 2014-11-03

**Authors:** Qiyi Luo, Xiangkun Liu, Zhonghua Li, Chubo Huang, Wen Zhang, Juan Meng, Zhaohua Chang, Zezhao Hua

**Affiliations:** 1 School of Medical Instrument and Food Engineering, University of Shanghai for Science and Technology, Shanghai, China; 2 Shanghai MicroPort Medical (Group) Co., Ltd., Shanghai, China; University of California, Irvine, United States of America

## Abstract

This study established a numerical model to investigate the degradation mechanism and behavior of bioabsorbable cardiovascular stents. In order to generate the constitutive degradation material model, the degradation characteristics were characterized with user-defined field variables. The radial strength bench test and analysis were used to verify the material model. In order to validate the numerical degradation model, in vitro bench test and in vivo implantation studies were conducted under physiological and normal conditions. The results showed that six months of degradation had not influenced the thermodynamic properties and mechanical integrity of the stent while the molecular weight of the stents implanted in the in vivo and in vitro models had decreased to 61.8% and 68.5% respectively after six month's implantation. It was also found that the degradation rate, critical locations and changes in diameter of the stents in the numerical model were in good consistency in both in vivo and in vitro studies. It implies that the numerical degradation model could provide useful physical insights and prediction of the stent degradation behavior and evaluate, to some extent, the in-vivo performance of the stent. This model could eventually be used for design and optimization of bioabsorbable stent.

## Introduction

Percutaneous Cardiovascular Intervention, PCI, has been widely used for the treatment of cardiovascular diseases because of its minimal trauma and high-efficiency [1]. With the developments in materials science and engineering technology, the overall performance of the Polycaprolactone (PCL) and Polylactide (PLA) bioabsorbable vascular stents (BVS) is getting closer to metal stents, where few reported clinical trials also confirmed the safety and efficacy of the BVS [2]–[5]. Moreover, the use of bioabsorbable stents reduces the risk of post-implantation side effects such late stent thrombosis and bleeding. BVS provides mechanical support for over a certain period of time and after the BVS degradation, the blood vessel recovers its normal functions while the BVS eventually absorbed by the body completely. The excellent biocompatibility and biodegradability of BVS show a great promise of alternative solution over the metal stent.

The in vitro hydrolytic degradation study of PCL [6], [7] and Poly-L-Lactide (PLLA) [8]–[10] has been previously been carried out. In the case of immiscible PLLA blends, it was found that the degradation rate of the polymers depended on the molecular weight, degree of crystallinity, crystal morphology as well as the phase microstructure of the material [11]. The alkaline products released from composite stent materials played an important role in the process of degradation. A previous study found that the alkaline products could provide a relatively steady environment for slowing down the degradation rate of PLLA while prolonging the degradation time of composite stents [12]. In a previous in vitro degradation report, it was found that PLLA stents degraded at a relatively slow rate, with their molecular weight decreasing linearly as a function of time [13]. The results of another study suggested that in the in vitro and in vivo environments, the degradation of PLLA rods proceeded at the same rate and followed the general sequence of aliphatic polyester degradation, ruling out enzymes contributing and accelerating the degradation rate in vivo [14].

The finite element method (FEM) is best known to find approximate solutions using numerical methods based on a boundary value. The finite element method is best understood when applied in practice as the finite element analysis (FEA). However, the serious lack of material constitutive models describing the degradation process of the bioabsorbable material hindered product design using FEA under complex loading conditions. So far, most of the research efforts on biodegradable polymers have been trial and error experiments [15]. Rajagopal et al. developed a strain-induced degradation model for polymeric solids [16]. The bioabsorbable material constitutive model was initially developed within the scope of the linearized theory of elasticity [17], [18], but was then extended to explain the nonlinear responses of finite deformations. João S. Soares et al. incorporated the bioabsorbable material constitutive model to a computerized system, where they calculated the mechanical responses of the three different stents during the degradation process [19].

The objective of this research is to study the degradation behavior and mechanism of bioabsorbable materials with in vivo experiments and theoretical analysis. The feasibility of the incorporation of such material models into the finite element analysis (FEA) of the behavior of the bioabsorbable stent materials was demonstrated and then, the design performance and clinical outcome of the bioabsorbable stents were determined by applying the models into practice. During this study, a thermodynamically consistent constitutive description of polymers with deformation-induced degradation was developed and applied during the analysis. We investigated and described the constitutive finite element analysis model of the bioabsorbable stents and the variations in the material degradation model by defining the degradation degree during the simulation of the degradation process of the stents. In order to validate the FEA degradation model, an in vivo model was also set up to investigate the degradation behavior and mechanism of the BVS.

## Materials and Methods

This research was performed on high molecular weight PLLA bioabsorbable cardiovascular stents. By investigating the variation trends of the in vivo and in vitro performances during the degradation process and by the using finite element analysis to simulate the degradation process, we expected to demonstrate the degradation mechanism of the bioabsorbable stent in the human body and hoped to complete more reliable designing and testing of the stent with the aid of in vitro test and numerical simulation method.

The experiment was carried out into two parts: In order to investigate the feasibility of the finite element method, the first part of the experiment consisted of the comparison between the finite element analysis and in vitro experiment, conducted at time points set at 10, 20 and 30 days; In order to assess the reliability of the in vitro experiment, the second part of the experiment consisted of the comparison between the in vitro and in vivo experiments, conducted at time points set at 1, 3 and 6 months.

The Polylactic Acid biodegradable coronary stent used in this study (manufactured by Shanghai MicroPort Medical (Group) Co. Ltd), was designed by using ultrafast laser cutting machine to cut PLLA tubing to achieve stent structure and then, the stent was crimped onto the surface of the balloon. The crimped stent was then delivered and released to the vascular lesions or mock artery model by means of the delivery system.

### *In vitro* model

**Figure 1:**
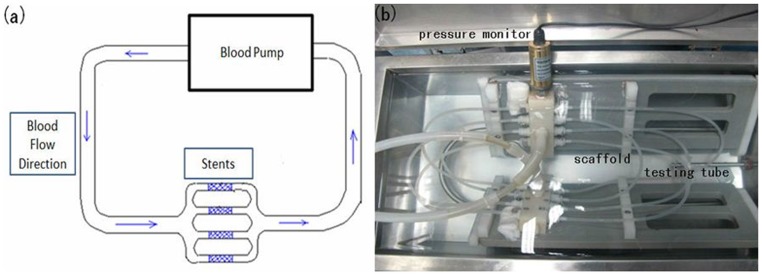
The in vitro simulation test setup. (a) Schematic of the in vitro degradation system; (b) picture of the in vitro degradation system.

The in vitro tests were carried out in a custom-made system simulating the in vivo environment which mainly comprised of mock artery loop and a plasma pump. The plasma pump provided the systolic/diastolic pressures and frequencies similar to the human circulatory system, where the systolic and diastolic pressures of the pump were set to 920 mmHg and 840 mmHg respectively (which resulted in 5% vascular compliance in the mock artery model). A fluid circulation of a frequency of 70 times/minute was set up, where phosphate buffer solution (PBS) was chosen to simulate the in vivo circulation medium. The PBS used for this study was sterilized for 20 minutes under high pressure at a temperature of 120°Cprior to usage. Antibacterial inhibitors that did not affect the sample and the degradation process were added to the solution. The circulation medium was replaced once every two weeks in order to maintain a pH value range of 7.4±0.2. The schematic diagram and the system are shown in Figure 1. When the stent was released into the artificial blood vessel of the in vitro model, the stent was subjected to a certain strain, which was necessary in order to prevent the blood vessel from shrinking.

**Figure 2:**
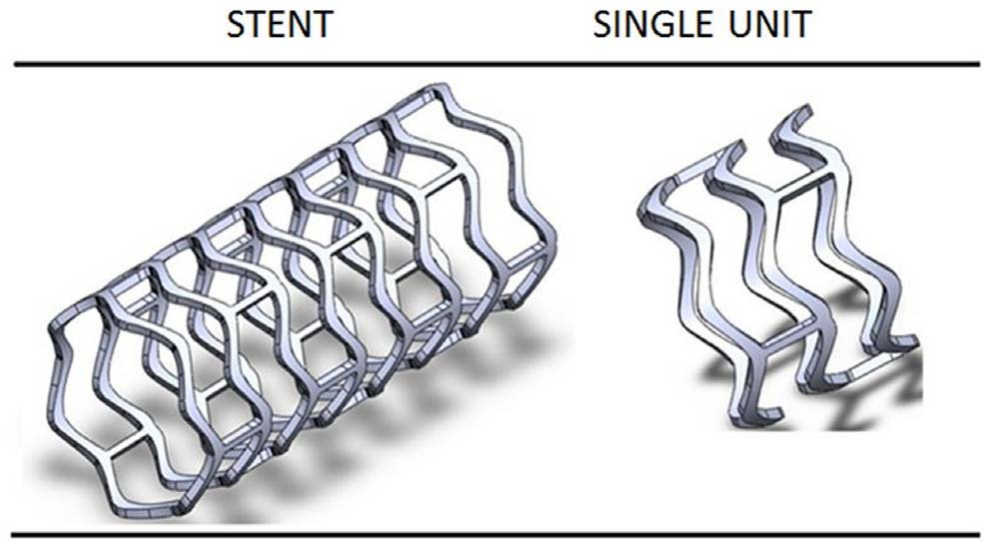
Stent model (left) and the single unit (right) used in the FEA simulation.

In this research, a new stent configuration was designed with the external diameter 3.5 mm and the thickness 0.125 mm. The stent design consisted of sinusoidal in-phase hoops linked by straight bridges. Figure 2 demonstrates the stent model in unexpanded configuration and the single unit used for the numerical simulations. The geometric model was created using SolidWorks 2011 (SolidWorks Corp., Concord, MA, USA).

### Numerical Model

The FE simulations of the stents were carried out using the FE program Simulia ABAQUS 6.10 (ABAQUS, Inc., Providence, RI, USA), where a mesh of 27232 3D eight-node brick element was generated (the element type C3D8I). The element type of the crimping tools and expansion tools used during the finite element analysis was surface element SFM3D4R.

The FEA analysis of bioabsorbable stents included six different analysis steps. The first step was the crimping simulation analysis, where a rigid cylindrical tube enacting the crimping tool was used to crimp the stent from an external diameter of 3.5 mm to 1.5 mm. After crimping, the stent was allowed to recoil freely by removing the rigid cylindrical tube, hereby simulating the elastic recoil after crimping. In the third step, another cylindrical rigid body, representing the balloon catheter, was used to make the stent expand to an internal diameter of 3.2 mm. Finally, in order to simulate the elastic recoil of the stent after expansion, the cylindrical rigid body was removed, allowing the stent to recoil freely.

During the fatigue analysis, the mock artery model was included [20]. In order to simulate the pulsatile fatigue, the physiological diastolic pressure P = 80 mmHg and systolic pressure P = 160 mmHg were applied to inner surface of the arterial wall. The dynamic and explicit user subroutine USDFLD was used to analyze the simulated degradation process. Different field variables were set by means of the user subroutine USDFLD of ABAQUS and by incorporating the material models at different conditions of degradation into the calculation, the variations in the material properties, such as the degradation degree, were determined. Eventually, the variation trends of the structure of the stent loaded during the degradation process were assessed.

During the radial strength analysis of the stent, a rigid cylindrical surface was used to crimp the stent and then the radial strength curve was obtained by means of in-house software post-processing.

The described analyses discussed the expansion performance of the bioabsorbable stent under the original tensile material property. After the implantation of the biodegradable stent in the human body, the stent initially provides support to the blood vessels and eventually completely disappears. During the degradation process, tensile tests were performed on the materials at different time points, hence analyzing the degradation procedure of the biodegradable materials.

**Figure 3:**
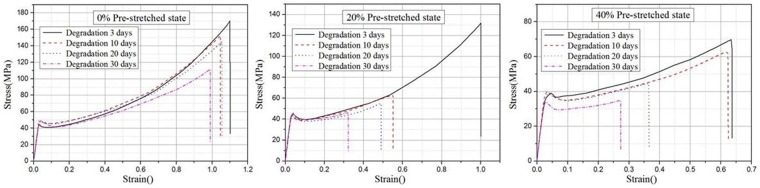
Stress-strain curves at different stretch magnitudes and degradation time.

As it can be seen from Figure 3, the biodegradable material has the following characteristics: The magnitude of the pre-stretch value and degradation time have very minor influences on the elastic modulus of the material. In general, the increase of the pre-stretched value and degradation time caused the decrease of the plastic curve. It is hereby specifically illustrated that under the same pre-stretched value, the longer the degradation time, the lower the plastic material curve and under the same degradation time, the greater the pre-stretched value, the lower the plastic material curve.

The USDFLD subroutine of ABAQUS/EXPLICIT was used to define effect of the pre-stretched value and degradation time on the degradation degree. The degradation degree hereby is defined as a user-defined field variable and is related to the elastic properties, plastic properties and fracture behavior of the material. The variable elastic/plastic properties and fracture behavior related to the different degradation degree are therefore obtained by ABAQUS linear interpolation.

The degradation degree, D, is defined as a transfer parameter of degradation level between different pre-stretched value and the degradation time. The range of degradation degree is from 0 to 1, where D = 0 represents the initial condition of the material prior to degradation while D = 1 represents the material at a state of complete degradation.

In this study, the degradation degree D is defined as the fracture elongation ratio of the material before and after degradation. The degradation degree D = D (strain, time) is defined as follows: According to the tensile tests curves of the material, Table 1 represents the elongation of the material at different degrees of degradation, where the value of the degradation degree D is calculated by obtaining the ratio of the elongation of the material after degradation and before degradation; The degradation degree of pre-stretched value 0% was assumed to be 0.1 after 3 days of degradation. Therefore, according to the linear relationship in Table 1, the degradation degree at the different degradation times was obtained. The exponential function used to describe the degradation degree D was: 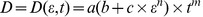
(1–1)


**Table 1:** Material property and degradation degree at different degradation times.

Variable		3 Days	10 Days	20 Days	30 Days
Pre-stretched 0%	Elastic Modulus(MPa)	1684.42	1590.85	1588.17	1606.68
	Elongation	1.09927	1.04615	1.0501	0.989781
	Degree	0.1	0.143491	0.140257	0.189641
Pre-stretched 20%	Elastic Modulus(MPa)	1621.48	1487.53	1515.71	1498.94
	Elongation	1.00152	0.555209	0.490494	0.321673
	Degree	0.18003	0.545436	0.59842	0.736638
Pre-stretched 40%	Elastic Modulus(MPa)	1575.15	1468.86	1514.06	1527.57
	Elongation	0.633277	0.619832	0.36437	0.272941
	Degree	0.48152	0.492528	0.701681	0.776536

There are five material constants in the formula, where the objective function was the deviation weighted sum between the fitting curve of degradation degree D and the test values. MATLAB Surface Fitting Tool was then used to fit the five material parameters (a, b, c, m, n) in the above described formula. The values of a, b, c, m and n obtained after fitting were 0.562, 0.2478, 1.094, 0.301 and 0.285 respectively and after including the 5 individual constants into the formula 1–1, the degradation degree formula obtained was:
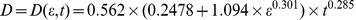
(1–2)


### *In vivo* Model

We declare that all the study submitted to PLOS ONE complies with the principles laid down in the Declaration of Helsinki. Animal research approved by Animal Care and Use Committee (IACUC) of Gateway Medical Innovation Center. In the in vivo experiments, pigs within a weight range of 32–32.5 kg were chosen for the stent degradation study. The implantation procedures were conducted by strictly following the clinical protocols, with the animal experiments complying to all applicable regulations. The stents were implanted into the coronary arteries of basically similar dimensions to the artificial blood vessels, that is the left anterior descending branch, the right coronary artery and the circumflex coronary artery. Three time points were chosen, that is 1, 3 and 6 months and two stents were implanted at each time point. In order to estimate the working conditions and performance of the stent, Optical coherence tomography (OCT) was used to measure the changes in the outer diameter of the in vivo stent. Pigs were sacrificed at the corresponding time points, with their hearts then perfused for 24 hours and placed in formalin solution. The stents were then retrieved and after removing attached intima, the stents were washed and dried under ambient temperature. Gel permeation chromatography (GPC) test was then performed on the stents. The purpose of GPC test was to analyze the molecular weight, where the molecular weight and content were determined by elution volume and concentration respectively. The GPC curve generated by elution volume and concentration, represented the polymer molecular weight distribution. In this study, the weight-average molecular weight and polydispersity index were analyzed.

## Results and Discussion

**Figure 4:**
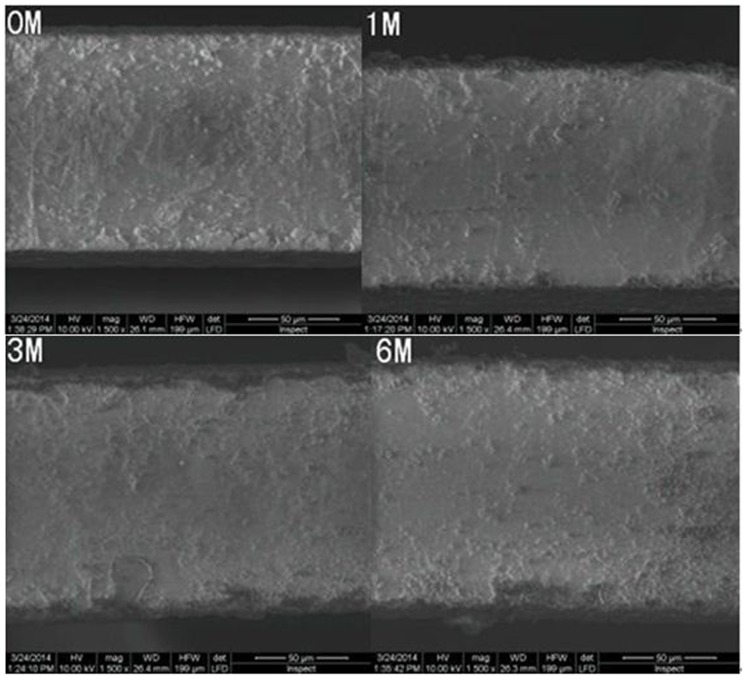
The changes in surface morphology of the stent in static and dynamic degradation system.

**Figure 5:**
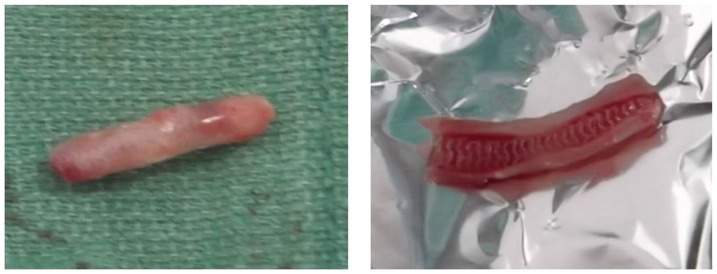
The stented anatomy of the in vivo model after 6-month implantation.

The degradation condition of the implanted stent of the in vivo models over the 6 months post-implantation can be observed from Figure 4, where it can be seen that the surface and edges of the stents from the in vitro degradation experiment suffered slight erosion, with a smaller area with thin skin fall. Yet, the stents were in overall good condition without any collapse phenomenon. Figure 5 demonstrates the anatomy of stent of the in vivo degradation model over the 6 months post-implantation: the implanted stent was able to keep good mechanical integrity and was successful in maintaining the support stability of blood vessels. It was seen that at 6 months after implantation, there was still minimal structure change in the stents in both the in vitro and in vivo models. The designed stent not only prevented extensive degradation during the six months, but could also support the patency of the blood vessels during 6 month implantation.

The outer diameter of the stents from the in vitro experiment was obtained by taking the average of the diameters at the proximal end, center and distal end of the stent, hereby accurately measured using a caliper. On the other hand, the outer diameter of the stents from the in vivo experiments was obtained by using a computer software to measure and fit the OTC images where the stent contour comprised of polylines arbitrarily connected by two perpendicular lines to the contour curve; the obtained calculated average length of the lines defined the outer diameter of the stent.

The radial strength of the stents in the in vitro experiment was measured by using the radial force tester MSI RX550 (Machine Solutions Inc). The time points for testing were set to 10, 20 and 30 days respectively and the experiment environment temperature was maintained to 37°C.

**Figure 6:**
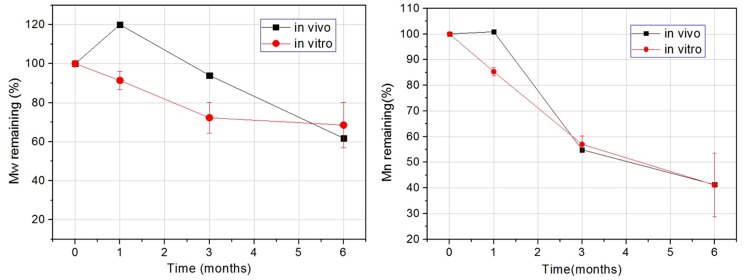
Change in the retention rate of the molecular weight of the stent material with respect to degradation time.

Figure 6 shows that the molecular weight of the stent decreased rapidly over the degradation time in both in vivo and in vitro models. Six months after implantation, the weight average molecular weight (Mw) of the in vivo and in vitro stents had respectively decreased to 61.8% and 68.5% of the initial molecular weight. As it can be seen in Figure 6, the decrease in the number average molecular weight (Mn) of the stents over the 0 to 6 months after implantation in the in vivo and in vitro models was consistent.

The molecular weight of the bioabsorbable material decreased along the process of degradation, but the loss in mass or the fracture of the material occurred only when the decrease in the molecular weight attained a certain threshold value. The degradation would start impacting the supporting performance of the stent only when the threshold value was attained. However, since this study only explored the early stages of degradation behavior; even though certain parts of the stent underwent accelerated degradation, the weight-averaged molecular weight and overall supporting performance of the stent were not significantly affected.

**Figure 7:**
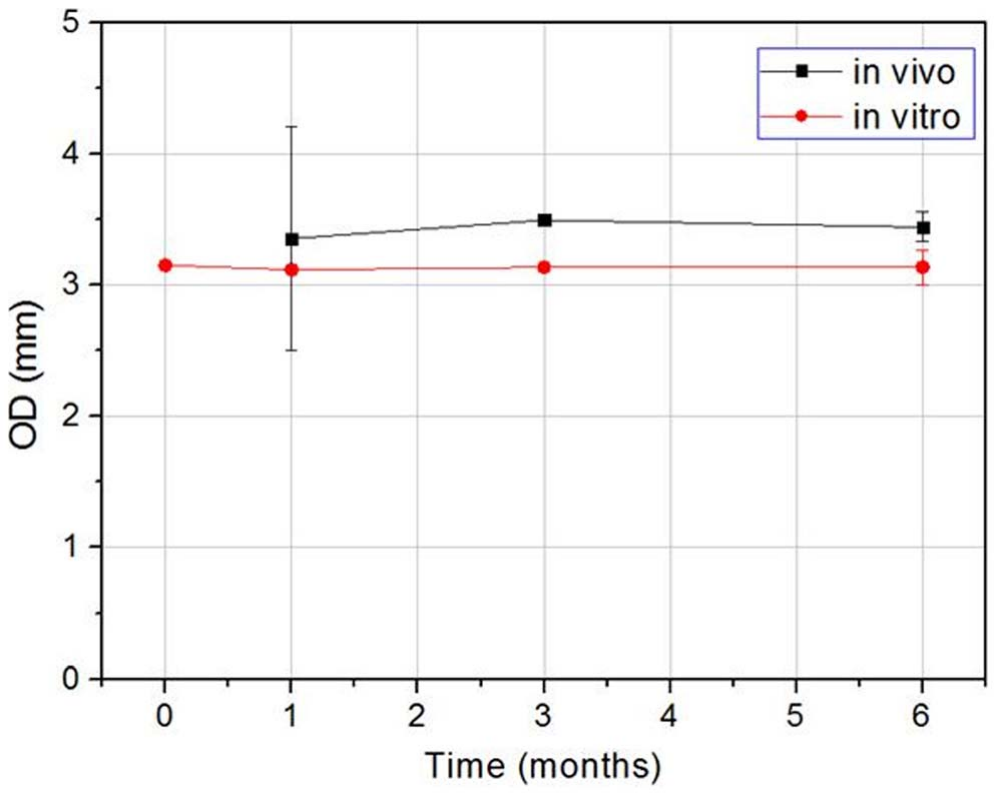
The stent diameter changes vs time of in vivo and in vitro models.

The outer diameters of the stent from the in vitro and in vivo models are illustrated in Figure 7. The stent diameter difference of in vivo and in vitro tests is mainly because of different measurement methods and the artery size variations between the in vitro and in vivo tests. Up to certain extent, the changes in the diameter of the stents reflected whether the stents had sufficient radial support and strength. The size of the stent did not reduce significantly over the degradation time, proving that when the stent was implanted into the blood vessels, it was able to function very well to support the blood vessels. It was also found that the change trend of the stent diameter was consistent in both in vitro and in vivo experiments. Hence, it was observed that during the in vivo and in vitro degradation process, the outer diameter of the implanted stent did not undergo significant changes over time, meaning that the stents were able to sustain appropriate radial strength.

**Figure 8:**
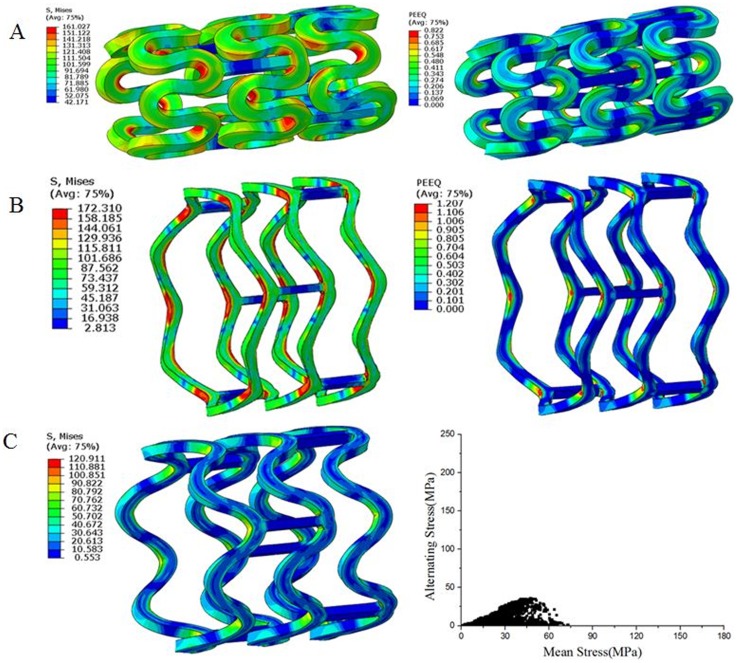
Stress and strain contours during the crimping analysis (A); Stress and strain contours during the expansion analysis (B); Stress and Goodman diagram during the stent fatigue analysis (C).

The initial properties of the bioabsorbable material prior to degradation were used to simulate the finite element analysis of the un-degraded stent. In the first step, the outer diameter of the stent was reduced to 1.5 mm by using a crimping tool, hence simulating the crimping analysis. From Figure 8, it can be seen that stress and strain were concentrated in the central region of the reinforcing unit of the stent. In the second step, the elastic recoil of the stent after crimping was simulated by removing the crimping tool; As it can be seen from the stress distribution, the stress in the stent decreased from 161 MPa to 102.4 MPa after the elastic recoil.

Eventually, after the crimping analysis, the stent was expanded. Figure 8 illustrates the stress and strain distributions of the stent when it was fully expanded and recoiled after expansion. As it can be seen from Figure 8, when the stent was fully expanded, the stress and strain were mainly concentrated at the central location, nearing the surface area of the inner curvature of the reinforced ring, where the tensile stress had reached 172.3 MPa with the strain value of 120.7%.

In the fatigue analysis, the physiological systolic pressure P = 160 mmHg was first applied to the inner surface of the arterial wall and then, the diastolic pressure P = 80 mmHg was applied to simulate the influence of the pulsatile blood pressure. The Goodman diagram [21]–[23] was used to illustrate the fatigue state of the stent while the distribution of the alternating stress points and average stress points are illustrated as in Figure 8.

**Figure 9:**
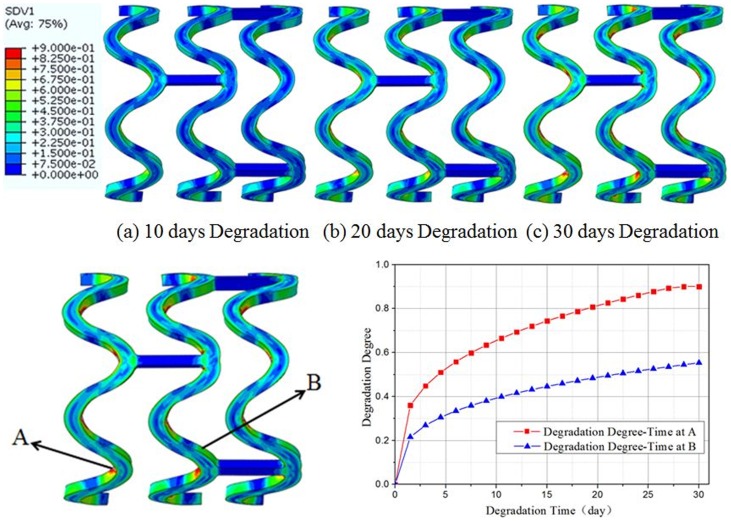
The degradation trends and the degradation degree vs. time at different positions of the stent.

The finite element model simulation of the degradation process was performed on the stent at an equilibrium state after the fatigue analysis, where the changes in the degradation state of the stent and the distribution of the degradation degree were analyzed. Figure 9 illustrates the changes in the degradation degree and the comparison of the degradation degree vs. time at different positions of the stent over 30 days of the degradation process. From the figure, it can be observed that after 30 days of degradation, the small area at the inner surface of the middle reinforced strut where the strain was initially larger, the degradation degree was maximum, stating that that area was almost in a state of complete degradation.

**Figure 10:**
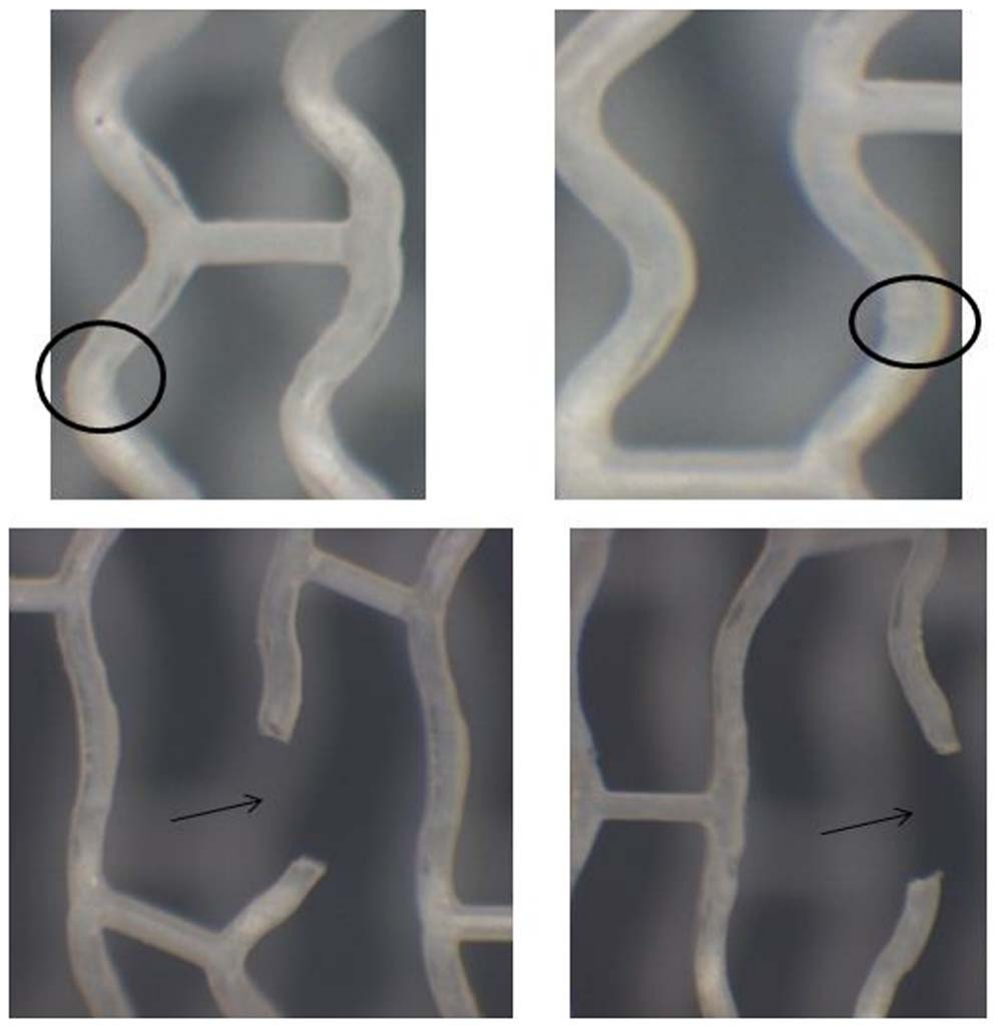
Stent strut thinning after six months of degradation and fracture after re-expansion.

As it can be seen from the Figures 9 and 10, the biodegradation process occurred across the entire stent. However, the degradation rates were not uniform in different locations along the stent structure: The material at the reinforced strut (the stent bridge) had the quickest degradation speed whereas the material away from that area had a relatively slower degradation speed. Therefore, it can be observed that the degradation speed of the material is related to the initial strain value of the material prior to degradation, where the greater the initial strain level, the faster the degradation speed. After 30 days of degradation, the small area at the inner surface of the middle reinforced strut with larger initial strain, had a maximum degradation degree of 0.9, implying that that area was almost in a state of complete degradation.

It was found that the stent degradation speed and location were closely related to the stress concentration on the stent after the implantation in the human body since the stents degraded at a relatively faster rate at the sites of stress concentration. When we further dilated the stents which were retrieved after the 6-month degradation, we found that all fracture sites were coincident with stress concentration regions with the faster rate of degradation. This study employed in vivo, in vitro and finite element analysis methods to investigate stent degradation and we found that the results of these methods were in good coherence with one another.

**Figure 11:**
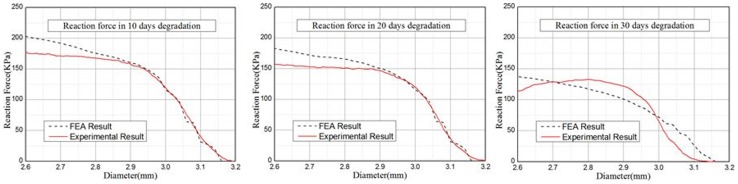
The comparison of the radial strength after 10 days (left), 20 days (middle) and 30 days (right) of degradation.

The radial strength analysis of the bioabsorbable stent was used to verify the accuracy of the biodegradation property model. From a macroscopic point of view, the radial strength curve of the bioabsorbable stent decreased to some extent. Figure 11 shows the comparison of the radial strength in the FE analysis and the experiments. The trends of the radial strength from FEA simulations and experiment tests at different degradation time points were in good coherence.

There are some factors causing the deviations in the FE analysis In the experimental data of the material degradation, only data of pre-stretch value ranging from 0% to 40% was covered, the experimental data with larger magnitude of pre-stretch was however not taken into consideration; Material properties are closely related to the degradation degree but still, the experimental data did not include the data about the properties of the almost completely degraded material; During the analysis process, since the material had degraded completely, its material property was obtained from the extrapolation of the experimental data, which eventually caused deviation in the results. Furthermore, the experimental data did not include the data about the material properties after complete degradation and this caused certain influence on the accuracy of the FE analysis. Also, the theory of the fatigue analysis method for the bioabsorbable stent still requires further research. In future research work, the above mentioned assumptions and limitations will be taken into consideration.

## Conclusions

In this study, the FE analysis method of the biodegradation process of bioabsorbable stents was developed. The accuracy of this model was validated by the in vivo and in vitro experiments and the radial strength bench test. The results of the in vivo and in vitro studies and the finite element analysis are in good accordance. The trends in the stent degradation speed, degradation location and changes in the diameter were highly consistent in all the experiments. Therefore, the results of the in vitro experiment and finite element analysis could be used as tools for guidance and reference during stent design and prediction of the in vivo degradation process, eventually reducing the cost of product development. It was also observed that even though degradation occurred at a faster rate at the reinforced strut of the stent where there was an increase in the low molecular weight chains, there was hereby limited impact on the weight-average molecular weight of the stent. The experiment results about the trends in the radial strength have proven that the weight-averaged molecular weight is a very important factor determining the supporting performance of the stent, with however limited influence on the radial supporting property. In summary, it is feasible to use the finite element model to predict the changes in the radial strength of bioabsorbable cardiovascular stents after degradation. However, in order to obtain more accurate analysis results, more comprehensive and accurate experimental data about the degradation and mechanical properties of the material are required.
